# Supplementary material for: Data encoding for healthcare data democratization and information leakage prevention
Source: Nat Commun. 2024 Feb 21;15:1582. doi: 10.1038/s41467-024-45777-z (PMC10882022; doi:10.1038/s41467-024-45777-z)
Supplement: Supplementary file 1 — Supplementary Information [file 41467_2024_45777_MOESM1_ESM.pdf]

Supplementary Information - Data Encoding For Healthcare Data Democratisation and Information Leakage Prevention

Supplementary Note 1 - Dataset Pre-processing

We have used the existing open source pipelines to obtain the pre-processed form of all datasets. For MIMIC-III, the benchmark[1] provided in [1] was used for obtaining the pre-processed time-series representing the ICU stays. FIDDLE pipeline[2] [2] was used to obtain the pre-processed version of the eICU dataset for the ARF prediction tasks.
Apart from that, we impute the missing values in PhysioNet dataset using *carry forward* approach[3].

Supplementary Note 2 - List of features in PhysioNet 2012 dataset

1. Alkaline phosphatase
2. Alanine transaminase
3. Aspartate transaminase
4. Albumin
5. Blood urea nitrogen
6. Bilirubin
7. Cholesterol
8. Creatinine
9. Invasive diastolic arterial blood pressure
10. Fractional inspired oxygen
11. Glasgow Comma Score
12. Glucose
13. Serum bicarbonate
14. Hematocrit
15. Heart rate
16. Height
17. ICU Type - Coronary Care Unit
18. ICU Type - Cardiac Surgery Recovery Unit
19. ICU Type - Medical ICU
20. ICU Type - Surgical ICU
21. Serum potassium
22. Lactate
23. Invasive mean arterial blood pressure
24. Mechanical ventilation respiration
25. Serum magnesium
26. Non-invasive diastolic arterial blood pressure

[1] github.com/YerevaNN/mimic3-benchmarks
[2] physionet.org/content/mimic-eicu-fiddle-feature/1.0.0/
[3] github.com/Ghadeer-Ghosheh/physionet2012-timeseries

27. Non-invasive mean arterial blood pressure
28. Non-invasive systolic arterial blood pressur
29. Serum sodium
30. Partial pressure of arterial CO_2
31. Partial pressure of arterial O_2
32. Platelets
33. Respiration rate
34. SAPS-I score
35. SOFA score
36. O_2 saturation in hemoglobin
37. Invasive systolic arterial blood pressure
38. Temperature
39. Troponin-I
40. Troponin-T
41. Urine output
42. White blood cell count
43. Weight
44. Arterial pH

Supplementary Note 3 - List of features in MIMIC-III dataset

1. Capillary refill rate-0.0
2. Capillary refill rate-1.0
3. Diastolic blood pressure
4. Fraction inspired oxygen
5. Glascow coma scale eye opening-2 To Pain
6. Glascow coma scale eye opening-3 To speech
7. Glascow coma scale eye opening-1 No Response
8. Glascow coma scale eye opening-4 Spontaneously
9. Glascow coma scale eye opening-0 None
10. Glascow coma scale motor response-1 No Movement
11. Glascow coma scale motor response-3 Abnormal flexion
12. Glascow coma scale motor response-2 Abnormal extension
13. Glascow coma scale motor response-4 Flex-withdraws
14. Glascow coma scale motor response-5 Localizes Pain
15. Glascow coma scale motor response-6 Obeys Commands
16. Glascow coma scale total-11
17. Glascow coma scale total-10
18. Glascow coma scale total-13
19. Glascow coma scale total-12
20. Glascow coma scale total-15
21. Glascow coma scale total-14
22. Glascow coma scale total-3
23. Glascow coma scale total-5
24. Glascow coma scale total-4
25. Glascow coma scale total-7
26. Glascow coma scale total-6
27. Glascow coma scale total-9
28. Glascow coma scale total-8
29. Glascow coma scale verbal response-1 No Response
30. Glascow coma scale verbal response-4 Confused
31. Glascow coma scale verbal response-2 Incomprehensible sounds
32. Glascow coma scale verbal response-3 Inappropriate Words
33. Glascow coma scale verbal response-5 Oriented
34. Glucose
35. Heart Rate
36. Height
37. Mean blood pressure
38. Oxygen saturation
39. Respiratory rate
40. Systolic blood pressure
41. Temperature
42. Weight
43. pH

44. mask-Capillary refill rate
45. mask-Diastolic blood pressure
46. mask-Fraction inspired oxygen
47. mask-Glascow coma scale eye opening
48. mask-Glascow coma scale motor response
49. mask-Glascow coma scale total
50. mask-Glascow coma scale verbal response
51. mask-Glucose
52. mask-Heart Rate
53. mask-Height
54. mask-Mean blood pressure
55. mask-Oxygen saturation
56. mask-Respiratory rate
57. mask-Systolic blood pressure
58. mask-Temperature
59. mask-Weight
60. mask-pH

Supplementary Note 4 - List of features used in the eICU dataset

1. Height: (0, 160.0]
2. Height: (160.0, 167.0]
3. Height: (167.0, 172.7]
4. Height: (172.7, 180.0]
5. Height: (180.0, 612.6]
6. Weight: (0.0, 63.0]
7. Weight: (63.0, 74.0]
8. Weight: (74.0, 85.0]
9. Weight: (85.0, 100.7]
10. Weight: (100.7, 953.0]
11. age: (17.999, 49.0]
12. age: (49.0, 60.0]
13. age: (60.0, 69.0]
14. age: (69.0, 78.0]
15. age: (78.0, 89.0]
16. age: >89
17. Airway type: No Artificial Airway
18. Apache Admission value: Acid-base/electrolyte disturbance
19. Apache Admission value: Angina, unstable
20. Apache Admission value: Bleeding, GI-location unknown
21. Apache Admission value: Bleeding, lower GI
22. Apache Admission value: Bleeding, upper GI
23. Apache Admission value: CHF, congestive heart failure
24. Apache Admission value: CVA, cerebrovascular accident
25. Apache Admission value: Coma/change in level of consciousness
26. Apache Admission value: Diabetic ketoacidosis
27. Apache Admission value: Embolus, pulmonary
28. Apache Admission value: Emphysema/bronchitis
29. Apache Admission value: Endarterectomy, carotid
30. Apache Admission value: Hemorrhage/hematoma, intracranial
31. Apache Admission value: Hypertension, uncontrolled
32. Apache Admission value: Infarction, acute myocardial
33. Apache Admission value: Pneumonia, bacterial
34. Apache Admission value: Renal failure, acute
35. Apache Admission value: Rhythm disturbance (atrial, supraventricular)
36. Apache Admission value: Rhythm disturbance (conduction defect)

37. Apache Admission value: Seizures (primary-no structural brain disease)
38. Apache Admission value: Sepsis, GI
39. Apache Admission value: Sepsis, cutaneous/soft tissue
40. Apache Admission value: Sepsis, pulmonary
41. Apache Admission value: Sepsis, renal/UTI (including bladder)
42. Apache Admission value: Sepsis, unknown
43. Hospital admit offset: (-529268.001, -1790.0]
44. Hospital admit offset: (-1790.0, -389.0]
45. Hospital admit offset: (-389.0, -173.0]
46. Hospital admit offset: (-173.0, -50.0]
47. Hospital admit offset: (-50.0, 118121.0]
48. Hospital admit source: Acute Care/Floor
49. Hospital admit source: Direct Admit
50. Hospital admit source: Emergency Department
51. Hospital admit source: Floor
52. Hospital admit source: Operating Room
53. Hospital admit source: Other Hospital
54. Hospital admit source: Recovery Room
55. Hospital admit source: Step-Down Unit (SDU)
56. Hospital Id: 110
57. Hospital Id: 122
58. Hospital Id: 141
59. Hospital Id: 148
60. Hospital Id: 165
61. Hospital Id: 167
62. Hospital Id: 171
63. Hospital Id: 176
64. Hospital Id: 183
65. Hospital Id: 199
66. Hospital Id: 208
67. Hospital Id: 243
68. Hospital Id: 252
69. Hospital Id: 264
70. Hospital Id: 281
71. Hospital Id: 283
72. Hospital Id: 300
73. Hospital Id: 307
74. Hospital Id: 331
75. Hospital Id: 338
76. Hospital Id: 365
77. Hospital Id: 394
78. Hospital Id: 411
79. Hospital Id: 413
80. Hospital Id: 417
81. Hospital Id: 420
82. Hospital Id: 435
83. Hospital Id: 443
84. Hospital Id: 458
85. Hospital Id: 73
86. Unit admit source: Acute Care/Floor
87. Unit admit source: Direct Admit
88. Unit admit source: Emergency Department
89. Unit admit source: Floor
90. Unit admit source: ICU
91. Unit admit source: ICU to SDU
92. Unit admit source: Operating Room
93. Unit admit source: Other Hospital
94. Unit admit source: Other ICU
95. Unit admit source: PACU
96. Unit admit source: Recovery Room
97. Unit admit source: Step-Down Unit (SDU)',
98. Unit stay type: admit
99. Unit stay type: readmit
100. Unit stay type: stepdown/other
101. Unit stay type: transfer

102. Unit type: CCU-CTICU
103. Unit type: CSICU
104. Unit type: CTICU
105. Unit type: Cardiac ICU
106. Unit type: MICU
107. Unit type: Med-Surg ICU
108. Unit type: Neuro ICU
109. Heart Rate mask
110. Non-Invasive BP Diastolic mask
111. Non-Invasive BP Systolic mask
112. O_2 Saturation mask
113. Respiratory Rate mask
114. CVP (-34.001, 5.0]
115. CVP (5.0, 8.0]
116. CVP (8.0, 11.0]
117. CVP (11.0, 15.0]
118. CVP (15.0, 396.0]
119. Invasive BP Diastolic (<48.0)
120. Invasive BP Diastolic (48.0, 56.0]
121. Invasive BP Diastolic (56.0, 62.0]
122. Invasive BP Diastolic (62.0, 71.0]
123. Invasive BP Diastolic (>71.0)
124. Invasive BP Mean (-50.001, 68.0]
125. Invasive BP Mean (68.0, 76.0]
126. Invasive BP Mean (76.0, 84.0]
127. Invasive BP Mean (84.0, 94.0]
128. Invasive BP Mean (>94.0)
129. Invasive BP Systolic (<104.0)
130. Invasive BP Systolic (104.0, 118.0]
131. Invasive BP Systolic (118.0, 131.0]
132. Invasive BP Systolic (131.0, 146.0]
133. Invasive BP Systolic (>146.0)
134. Non-Invasive BP Mean (<67.0)
135. Non-Invasive BP Mean (67.0, 76.0]
136. Non-Invasive BP Mean (76.0, 84.0]
137. Non-Invasive BP Mean (84.0, 95.0]
138. Non-Invasive BP Mean (>95.0)
139. O_2 Admin Device: BiPAP
140. O_2 Admin Device: BiPAP/C-PAP
141. O_2 Admin Device: NC
142. O_2 Admin Device: RA
143. O_2 Admin Device: nasal cannula
144. O_2 Admin Device: nc
145. O_2 Admin Device: non-rebreather
146. O_2 Admin Device: other
147. O_2 Admin Device: ra
148. O_2 Admin Device: room air
149. O_2 Admin Device: trach collar
150. O_2 Admin Device: ventilator
151. O_2 Admin Device: venturi mask
152. O_2 L%: (<2.0)
153. O_2 L%: (2.0, 3.0]
154. O_2 L%: (3.0, 6.0]
155. O_2 L%: >6.0
156. Temperature (C): (<36.4)
157. Temperature (C): (36.4, 36.7]
158. Temperature (C): (36.7, 36.9]
159. Temperature (C): (36.9, 37.2]
160. Temperature (C): (>37.2)
161. Temperature (F): (<97.5)
162. Temperature (F): (97.5, 98.1]
163. Temperature (F): (98.1, 98.4]
164. Temperature (F): (98.4, 99.0]
165. Temperature (F): (>99.0)
166. Temperature Location: (-0.001, 1.0]
167. Temperature Location: (1.0, 4.0]
168. Temperature Location: TA
169. Temperature Location: AXILLARY
170. Temperature Location: BLADDER
171. Temperature Location: Core urinary catheter
172. Temperature Location: Forehead
173. Temperature Location: Oral
174. Temperature Location: PA CATHETER
175. Temperature Location: Rectal
176. Temperature Location: Skin Sensor
177. Temperature Location: TEMPORAL

178. Temperature Location: TEMPORAL ARTERY
179. Temperature Location: TYMPANIC
180. Temperature Location: Temporal Artery Scan
181. Temperature Location: Temporal scan
182. Temperature Location: core
183. Temperature Location: undocumented
184. Non-Invasive BP Diastolic delta time: (-0.001, 1.0]
185. Non-Invasive BP Diastolic delta time: (-0.001, 1.0]
186. Heart Rate: (-0.001, 68.0]
187. Heart Rate: (68.0, 78.0]
188. Heart Rate: (78.0, 88.0]
189. Heart Rate: (88.0, 100.0]
190. Heart Rate: (100.0, 300.0]
191. Non-Invasive BP Diastolic: (-0.001, 54.0]
192. Non-Invasive BP Diastolic: (54.0, 61.0]
193. Non-Invasive BP Diastolic: (61.0, 69.0]
194. Non-Invasive BP Diastolic: (69.0, 79.0]
195. Non-Invasive BP Diastolic: (79.0, 866.0]
196. Non-Invasive BP Systolic: (-0.001, 102.0]
197. Non-Invasive BP Diastolic: (102.0, 114.0]
198. Non-Invasive BP Diastolic: (114.0, 127.0]
199. Non-Invasive BP Systolic: (127.0, 142.0]
200. Non-Invasive BP Diastolic: (142.0, 12065.0]
201. O_2 Saturation: (-0.001, 95.0]
202. O_2 Saturation: (95.0, 96.0]
203. O_2 Saturation: (96.0, 98.0]
204. O_2 Saturation: (98.0, 99.0]
205. O_2 Saturation: (99.0, 999.0]
206. Respiratory Rate: (-0.001, 15.0]
207. Respiratory Rate: (15.0, 17.0]
208. Respiratory Rate: (17.0, 20.0]
209. Respiratory Rate: (20.0, 24.0]
210. Respiratory Rate: (24.0, 912.0]
211. Heart Rate min: (-0.001, 67.0]
212. Heart Rate min: (67.0, 77.0]
213. Heart Rate min: (77.0, 87.0]
214. Heart Rate min: (87.0, 99.0]
215. Heart Rate min: (99.0, 293.0]
216. Heart Rate max: (-0.001, 69.0]
217. Heart Rate max: (69.0, 79.0]
218. Heart Rate max: (79.0, 88.0]
219. Heart Rate max: (88.0, 101.0]
220. Heart Rate max: (101.0, 959.0]
221. Heart Rate mean: (-0.001, 68.0]
222. Heart Rate mean: (68.0, 78.0]
223. Heart Rate mean: (78.0, 87.5]
224. Heart Rate mean: (87.5, 100.0]
225. Heart Rate mean: (100.0, 527.0]
226. Non-Invasive BP Diastolic min: (-0.001, 52.0]
227. Non-Invasive BP Diastolic min: (52.0, 60.0]
228. Non-Invasive BP Diastolic min: (60.0, 68.0]
229. Non-Invasive BP Diastolic min: (68.0, 78.0]
230. Non-Invasive BP Diastolic min: (78.0, 777.0]
231. Non-Invasive BP Diastolic max: (-0.001, 55.0]
232. Non-Invasive BP Diastolic max: (55.0, 63.0]
233. Non-Invasive BP Diastolic max: (63.0, 70.0]
234. Non-Invasive BP Diastolic max: (70.0, 80.0]
235. Non-Invasive BP Diastolic max: (80.0, 6078.0]
236. Non-Invasive BP Diastolic mean: (-0.001, 54.0]
237. Non-Invasive BP Diastolic mean: (54.0, 61.25]

238. Non-Invasive BP Diastolic mean: (61.25, 69.0]
239. Non-Invasive BP Diastolic mean: (69.0, 79.0]
240. Non-Invasive BP Diastolic mean: (79.0, 1578.5]
241. Non-Invasive BP Systolic min: (-0.001, 100.0]
242. Non-Invasive BP Systolic min: (100.0, 113.0]
243. Non-Invasive BP Systolic min:(113.0, 125.0]
244. Non-Invasive BP Systolic min: (125.0, 141.0]
245. Non-Invasive BP Systolic min: (141.0, 12065.0]
246. Non-Invasive BP Systolic max: (-0.001, 104.0]
247. Non-Invasive BP Systolic max: (104.0, 116.0]
248. Non-Invasive BP Systolic max: (116.0, 128.0]
249. Non-Invasive BP Systolic max: (128.0, 144.0]
250. Non-Invasive BP Systolic max: (144.0, 12065.0]
251. Non-Invasive BP Systolic mean: (-0.001, 102.0]
252. Non-Invasive BP Systolic mean: (102.0, 114.0]
253. Non-Invasive BP Systolic mean: (114.0, 126.5]
254. Non-Invasive BP Systolic mean: (126.5, 142.0]
255. Non-Invasive BP Systolic mean: (142.0, 12065.0]
256. O_2 Saturation min: (-0.001, 94.0]
257. O_2 Saturation min: (94.0, 96.0]
258. O_2 Saturation min: (96.0, 98.0]
259. O_2 Saturation min: (98.0, 99.0]
260. O_2 Saturation min: (99.0, 999.0]
261. O_2 Saturation max: (-0.001, 95.0]
262. O_2 Saturation max: (95.0, 97.0]
263. O_2 Saturation max: (97.0, 98.0]
264. O_2 Saturation max: (98.0, 100.0]
265. O_2 Saturation mean: (-0.001, 95.0]
266. O_2 Saturation mean: (95.0, 96.0]
267. O_2 Saturation mean: (96.0, 98.0]
268. O_2 Saturation mean: (98.0, 99.0]
269. O_2 Saturation mean: (99.0, 999.0]
270. Respiratory Rate min: (-0.001, 14.0]
271. Respiratory Rate min: (14.0, 17.0]
272. Respiratory Rate min: (17.0, 19.0]
273. Respiratory Rate min: (19.0, 23.0]
274. Respiratory Rate min: (23.0, 912.0]
275. Respiratory Rate max: (-0.001, 15.0]
276. Respiratory Rate max: (15.0, 18.0]
277. Respiratory Rate max: (18.0, 20.0]
278. Respiratory Rate max: (20.0, 24.0]
279. Respiratory Rate max: (24.0, 2122.0]
280. Respiratory Rate mean: (-0.001, 15.0]
281. Respiratory Rate mean: (15.0, 17.667]
282. Respiratory Rate mean: (17.667, 20.0]
283. Respiratory Rate mean: (20.0, 23.25]
284. Respiratory Rate mean: (23.25, 912.0]

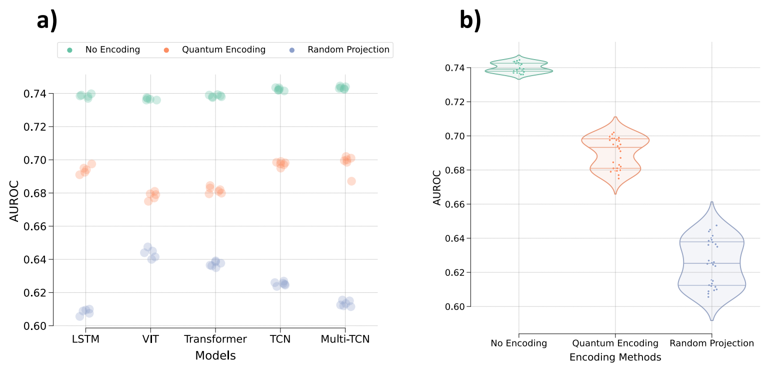

Supplementary Figure S1: **(a)** Performance of LSTM, vision transformer (ViT), transformer, temporal convolutional network (TCN) and multi-branch temporal convolutional network (Multi-TCN) for phenotype prediction. **(b)** Aggregate performance of all models as a function of encoding method. Source data are provided as a Source Data file.

Supplementary Note 5 - List of phenotypes or patient disorders in MIMIC-III dataset

1. Acute and unspecified renal failure
2. Acute cerebrovascular disease
3. Acute myocardial infarction
4. Cardiac dysrhythmias
5. Chronic kidney disease
6. Chronic obstructive pulmonary disease
7. Complications of surgical/medical care
8. Conduction disorders
9. Congestive heart failure; nonhypertensive
10. Coronary atherosclerosis and related
11. Diabetes mellitus with complications
12. Diabetes mellitus without complication
13. Disorders of lipid metabolism
14. Essential hypertension
15. Fluid and electrolyte disorders
16. Gastrointestinal hemorrhage
17. Hypertension with complications
18. Other liver diseases
19. Other lower respiratory disease
20. Other upper respiratory disease
21. Pleurisy; pneumothorax; pulmonary collapse
22. Pneumonia
23. Respiratory failure; insufficiency; arrest
24. Septicemia (except in labor)
25. Shock

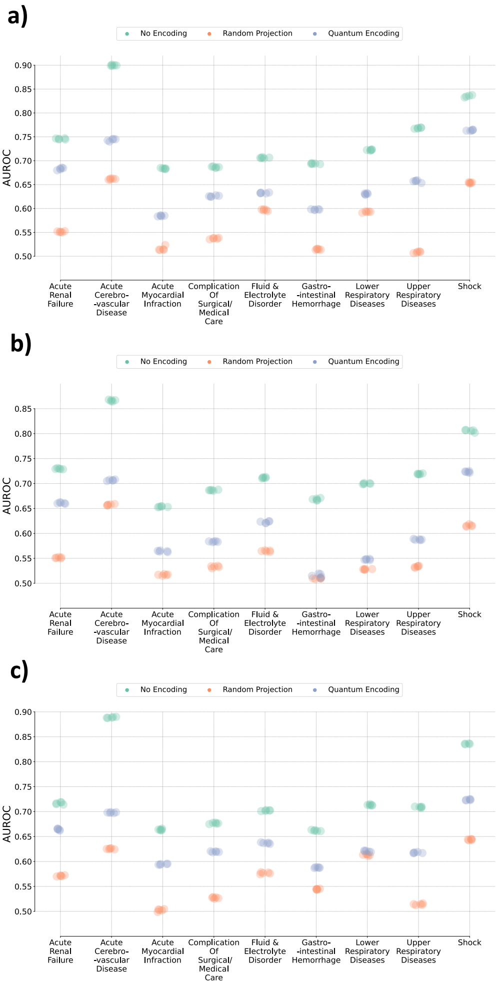

Supplementary Figure S2: Latent acute disorder prediction from **(a)** transformer, **(b)** temporal convolutional network and **(c)** multi-branch temporal convolutional network trained for mortality prediction. Source data are provided as a Source Data file.

Supplementary Note 6 - Performance on the encoded data for the task of Phenotyping

As discussed in Results (main document), along with mortality labels, we also have information about the 25 phenotypes or disorders corresponding to each ICU stay. In the main text, we used these disorders for evaluating the information leakage from the trained mortality prediction models using a multi-label multi-class setup.

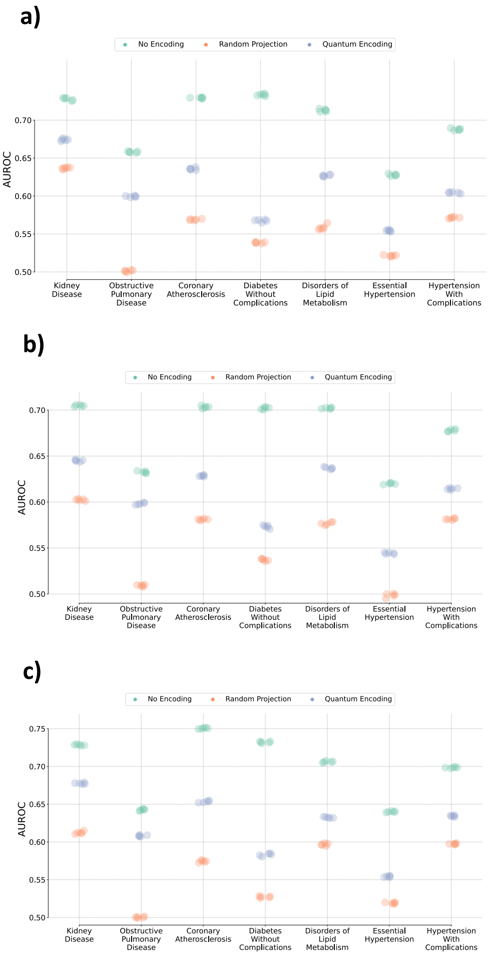

Supplementary Figure S3: Latent chronic disorder prediction from **(a)** transformer, **(b)** temporal convolutional network and **(c)** multi-branch temporal convolutional network trained for mortality prediction. Source data are provided as a Source Data file.

Here, we train all the models discussed in the main text for directly phenotyping each ICU stay. The last layers of these models were changed to have 25 nodes followed by sigmoid activation to favour multi-label multi-class predictions. The train, test and validation setup used for mortality prediction is also used here. Adam optimiser with a learning of 0.001 and a batch-size of 64 is used for training all models.

Fig. S1 illustrates the performance of different models as a function of encoding method for the task of phenotyping. Similar to the binary prediction experiments, the relation between encoding methods and model performance

is almost identical. Both random projection and quantum encoding results in a noticeable drop in performance. However, this drop is much more bearable in the case of quantum encoding.

Supplementary Note 7 - Latent prediction of chronic and acute disorders from all models

Fig. 5 of the main text shows the accuracy in prediction of different chronic and acute disorders from LSTM mortality prediction models. Similarly, Fig. S2 and Fig. S3 document the performance of predicting chronic and acute disorders from other mortality prediction models.

Supplementary Note 8 - Odds Ratio: Acute and Chronic Conditions vs Mortality in MIMIC-III dataset

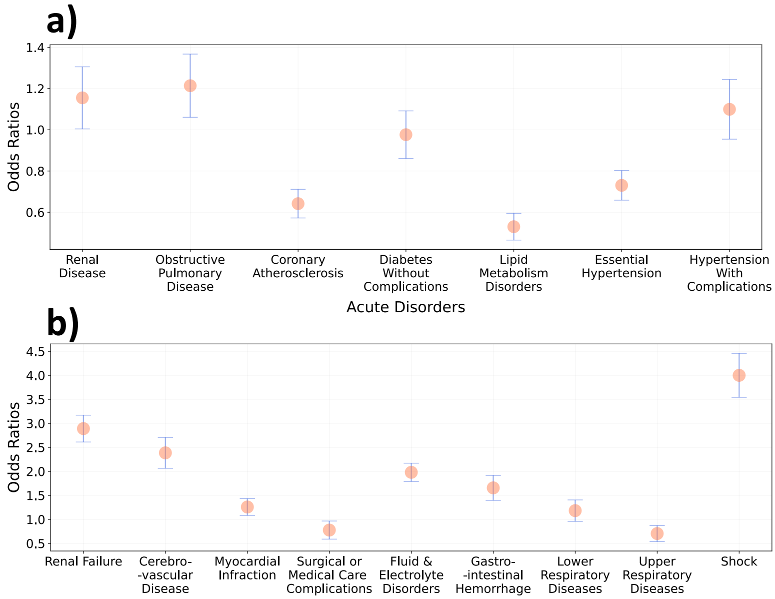

Supplementary Figure S4: Odds ratios comparing the presence of **a)** acute disorders and mortality, along with **b)** chronic disorders and mortality within the MIMIC-III dataset. The error bars represent standard deviation of odd ratios. All positive examples ($n = 2799$) in MIMIC-III dataset are used for computing odd ratios. Source data are provided as a Source Data file.

Supplementary Note 9 - Odds Ratio: Ethnicity vs Acute Respiration Failure in eICU dataset

The analysis of Fig. S5 highlights the odds ratio for *Black*, *Asian* and *Caucasian* are close to 1. This shows that there is no apparent association between ethnicity and ARF in the eICU dataset. Despite that, we are able to predict the ethnicity of the patients from trained ARF models effectively (Fig 4 of the manuscript).

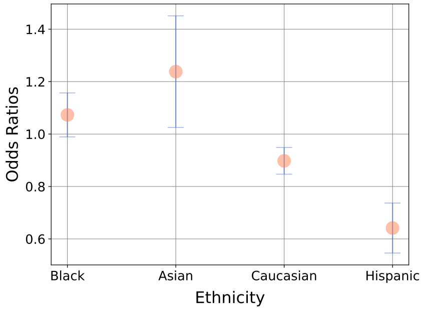

Supplementary Figure S5: Odds ratios of different ethnicity to the acute respiratory failure in the eICU dataset. The error bars represent standard deviation of odd ratios. All positive examples ($n = 57434$) in eICU dataset are used for computing odd ratios. Source data are provided as a Source Data file.

Supplementary Note 10 - Visual inspection of encoded PhysioNet examples

Fig. S6 illustrates the differences in encoded and original time-series examples from PhysioNet dataset.

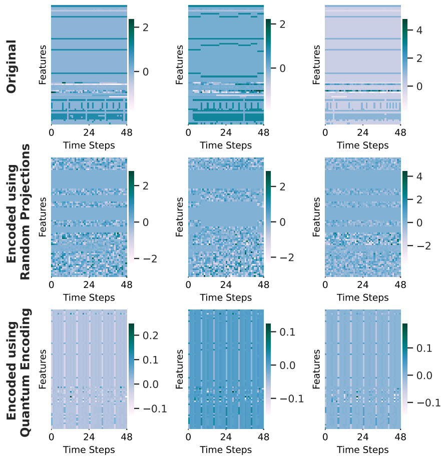

(a) Three negative examples from MIMIC-III.

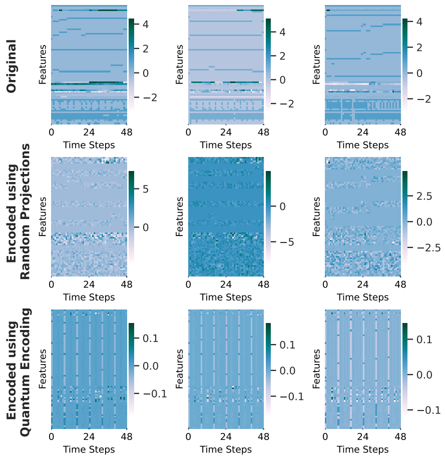

(b) Three positive examples from MIMIC-III.

Supplementary Figure S6: Heat maps illustrating the differences in magnitude and trends of the original and encoded time-series examples. Each row represents an input time series and its encoded versions. In each heat map, x-axis represents the time-steps while y-axis represents the 60 features in MIMIC-III dataset.

Supplementary Note 11 - Predicting Gender and Ethnicity from Encoded and Raw Datasets

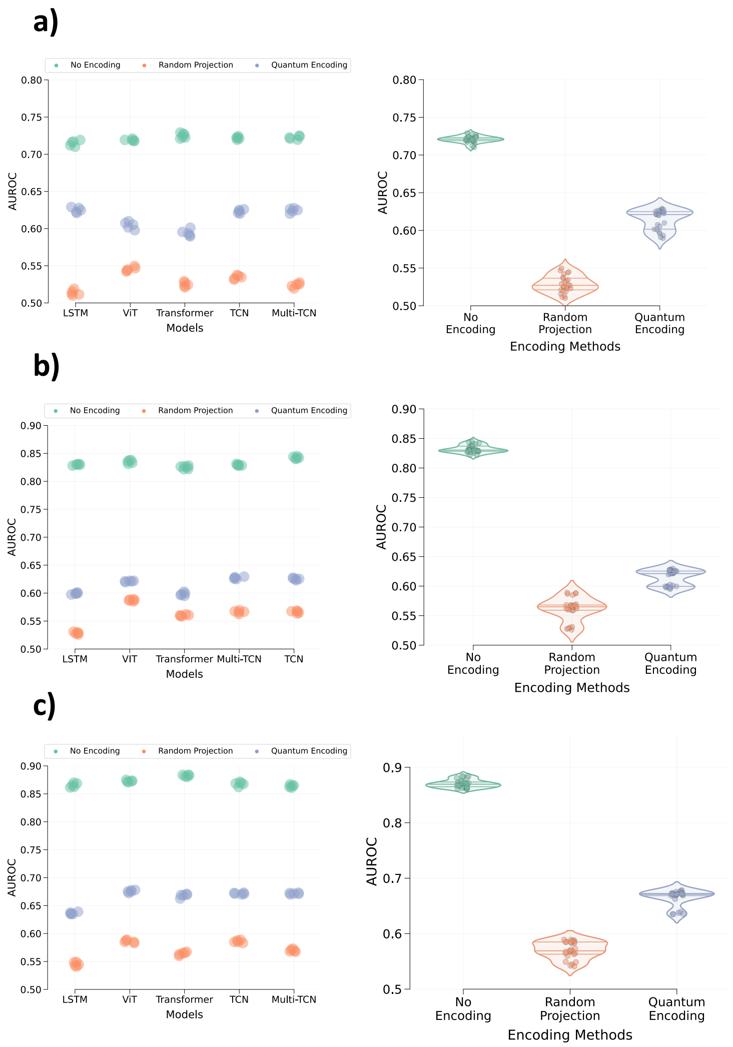

Supplementary Figure S7: Performance of LSTM, vision transformer (ViT), transformer, temporal convolutional network (TCN) and multi-branch temporal convolutional network (Multi-TCN) for predicting gender from raw time-series examples in **(a)** MIMIC-III, **(b)** PhysioNet and **(c)** eICU datasets, respectively. The violin plots in second column illustrate the gender prediction performance as a function of encoding methods across different models on each dataset. Source data are provided as a Source Data file.

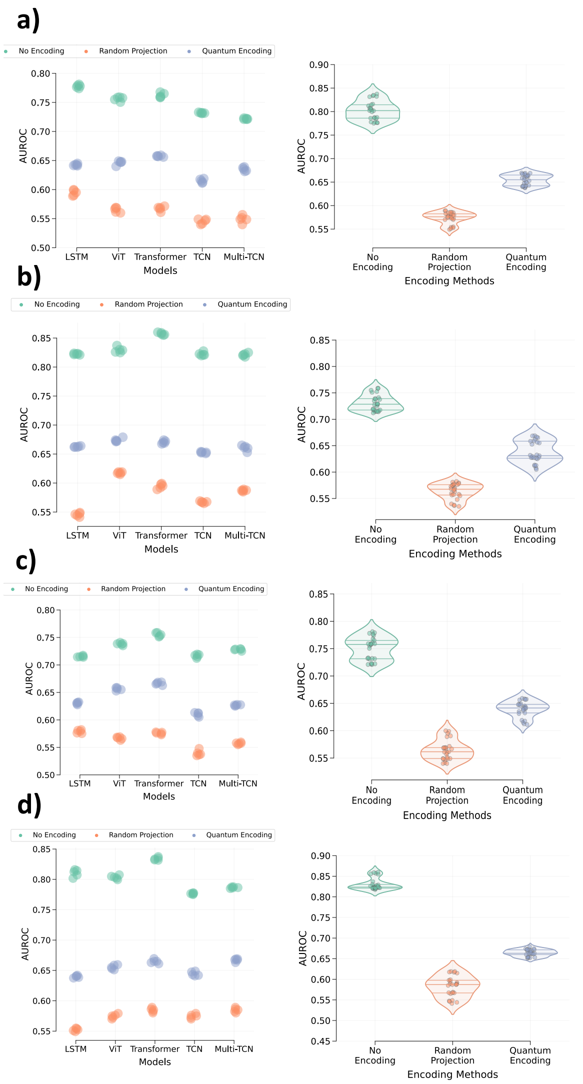

Supplementary Figure S8: Performance of LSTM, vision transformer (ViT), transformer, temporal convolutional network (TCN) and multi-branch temporal convolutional network (Multi-TCN) for predicting if a patient is **(a)** African-American, **(b)** Hispanic, **(c)** Caucasian and **(d)** Asian, respectively. The violin plots in second column illustrate the ethnicity prediction performance as a function of encoding methods across different models. Source data are provided as a Source Data file.

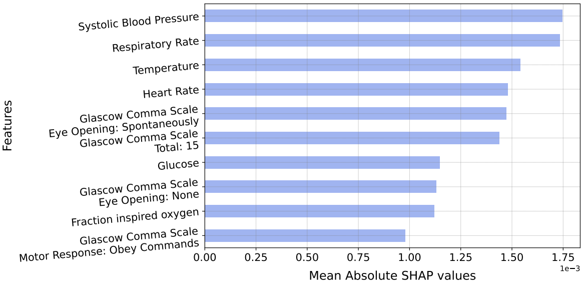

(a) SHAP analysis of LSTM trained on MIMIC-III.

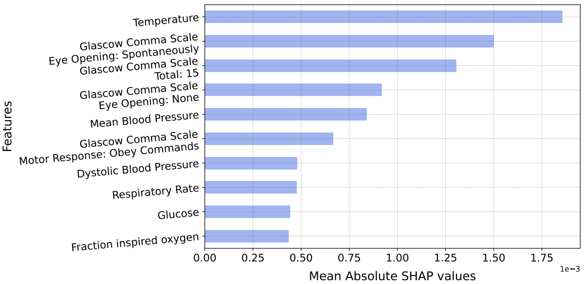

(b) SHAP analysis of LSTM trained on quantum encoded MIMIC-III.

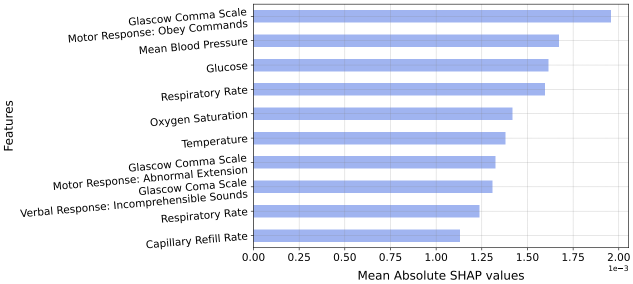

(c) SHAP analysis of LSTM trained on MIMIC-III data encoded by random projections.

Supplementary Figure S9: A comparison of SHAP-based feature importance in LSTM models trained on (a) original, (b) quantum encoded, and (c) randomly projected versions of the MIMIC-III dataset. Source data are provided as a Source Data file.

Supplementary Note 12 - SHAP Analysis of LSTM models trained on MIMIC-III

Fig.S9 illustrates SHAP plots for LSTM models trained on original and encoded MIMIC-III data.

Supplementary Table S1: Effect of the number of attention layers in Transformers trained for mortality prediction on MIMIC-III dataset.

**Depth**	**#Parameters (millions)**	**Encoding**					
		No Encoding		***Quantum***		***Random Projection***	
		Mortality	*Gender*	*Mortality*	*Gender*	*Mortality*	*Gender*
1	1.25	**0.838** ± 0.001	**0.709** ± 0.002	**0.816** ± 0.002	0.551 ± 0.001	**0.719** ± 0.001	**0.523** ± 0.002
2	2.51	0.831 ± 0.003	0.704 ± 0.002	0.811 ± 0.003	**0.552** ± 0.002	0.718 ± 0.002	**0.523** ± 0.001
4	5.01	0.826 ± 0.003	0.7 ± 0.001	0.813 ± 0.002	0.549 ± 0.001	0.717 ± 0.002	0.522 ± 0.002
8	10.1	0.827 ± 0.002	0.697 ± 0.002	0.812 ± 0.002	0.55 ± 0.002	0.717 ± 0.001	0.521 ± 0.002
16	20.21	0.826 ± 0.002	0.699 ± 0.002	0.813 ± 0.002	0.551 ± 0.001	0.717 ± 0.002	0.522 ± 0.001

Supplementary Table S2: Effect of the number of attention layers in Transformers trained for mortality prediction on PhysioNet dataset.

**Depth**	**#Parameters (millions)**	**Encoding**					
		No Encoding		***Quantum***		***Random Projection***	
		Mortality	*Gender*	*Mortality*	*Gender*	*Mortality*	*Gender*
1	1.25	**0.833** ± 0.002	**0.756** ± 0.002	0.772 ± 0.003	**0.578** ± 0.002	0.689 ± 0.003	**0.527** ± 0.002
2	2.51	**0.833** ± 0.003	0.755 ± 0.002	**0.773** ± 0.002	0.577 ± 0.003	0.687 ± 0.001	**0.527** ± 0.001
4	5.01	0.829 ± 0.002	0.753 ± 0.002	0.772 ± 0.001	0.575 ± 0.002	0.687 ± 0.002	0.526 ± 0.003
8	10.1	0.828 ± 0.001	0.754 ± 0.001	0.771 ± 0.003	0.576 ± 0.003	0.688 ± 0.002	0.525 ± 0.001
16	20.21	0.829 ± 0.001	0.753 ± 0.002	0.772 ± 0.002	0.576 ± 0.002	0.688 ± 0.003	0.526 ± 0.001

Supplementary Table S3: Effect of the number of attention layers in Transformers trained for acute respiratory failure (ARF) prediction on the eICU dataset.

**Depth**	**#Parameters (millions)**	**Encoding**					
		No Encoding		***Quantum***		***Random Projection***	
		Mortality	*Gender*	*Mortality*	*Gender*	*Mortality*	*Gender*
1	1.25	**0.766** ± 0.004	**0.908** ± 0.003	0.753 ± 0.002	**0.701** ± 0.003	0.698 ± 0.003	0.589 ± 0.004
2	2.51	0.762 ± 0.004	0.904 ± 0.005	**0.756** ± 0.004	**0.701** ± 0.004	0.695 ± 0.004	**0.591** ± 0.003
4	5.01	0.765 ± 0.003	0.899 ± 0.005	0.754 ± 0.004	0.698 ± 0.004	0.691 ± 0.004	0.587 ± 0.005
8	10.1	0.763 ± 0.004	0.905 ± 0.003	0.751 ± 0.004	0.7 ± 0.005	0.696 ± 0.005	0.59 ± 0.003
16	20.21	0.765 ± 0.003	0.906 ± 0.004	0.755 ± 0.004	0.697 ± 0.004	**0.699** ± 0.004	0.588 ± 0.003

Supplementary Note 14 - Impact of model complexity and latent prediction tasks

To prove that the proposed encoding framework can withstand deeper or complex models, we repeated the latent gender prediction experiment with larger transformers and ViTs. We varied the number of attention layers from 1 to 2, 4, 8 and 16 to analyse the impact on latent predictions. The results for transformers on MIMIC, PhysioNet and eICU are documented in Tables S1, S2 and S3, respectively. The analysis of these tables shows that no significant improvement is observed on both targeted (mortality prediction) as well as the latent (gender) prediction tasks as we increase the number of attention layers. Moreover, in some cases, we noticed a minute drop as model complexity is increased. Similar trends are observed for ViT as documented in Tables S4, S5 and S6.

Supplementary Table S4: Effect of the number of attention layers in ViT trained for mortality prediction on MIMIC-III dataset.

Depth	#Parameters (millions)	Encoding					
		No Encoding		***Quantum***		***Random Projection***	
		Mortality	*Gender*	*Mortality*	*Gender*	*Mortality*	*Gender*
1	1.25	**0.833** ± 0.003	**0.689** ± 0.003	**0.772** ± 0.003	0.546 ± 0.001	**0.714** ± 0.004	**0.493** ± 0.002
2	2.51	0.832 ± 0.003	**0.689** ± 0.005	0.77 ± 0.004	**0.547** ± 0.002	0.711 ± 0.002	**0.493** ± 0.003
4	5.01	0.828 ± 0.004	0.688 ± 0.003	0.769 ± 0.003	0.545 ± 0.004	0.709 ± 0.003	0.489 ± 0.003
8	10.1	0.829 ± 0.004	0.687 ± 0.003	0.769 ± 0.003	**0.547** ± 0.003	0.71 ± 0.003	0.488 ± 0.002
16	20.21	0.826 ± 0.002	0.688 ± 0.002	0.767 ± 0.004	0.546 ± 0.003	0.712 ± 0.002	0.492 ± 0.003

Supplementary Table S5: Effect of the number of attention layers in ViT trained for mortality prediction on PhysioNet dataset.

Depth	#Parameters (millions)	Encoding					
		No Encoding		***Quantum***		***Random Projection***	
		Mortality	*Gender*	*Mortality*	*Gender*	*Mortality*	*Gender*
1	1.25	**0.837** ± 0.003	**0.711** ± 0.003	**0.769** ± 0.003	**0.563** ± 0.003	**0.653** ± 0.003	0.552 ± 0.003
2	2.51	0.836 ± 0.004	0.71 ± 0.003	0.766 ± 0.004	0.561 ± 0.004	**0.653** ± 0.003	**0.554** ± 0.001
4	5.01	0.836 ± 0.003	0.71 ± 0.004	0.767 ± 0.002	0.562 ± 0.004	0.651 ± 0.004	0.551 ± 0.003
8	10.1	0.833 ± 0.003	0.708 ± 0.003	0.765 ± 0.004	0.559 ± 0.002	0.648 ± 0.004	0.55 ± 0.004
16	20.21	0.834 ± 0.004	0.709 ± 0.004	0.762 ± 0.005	0.561 ± 0.003	0.649 ± 0.004	0.549 ± 0.003

Supplementary Table S6: Effect of the number of attention layers in ViT trained for acute respiratory failure (ARF) prediction on the eICU dataset.

Depth	#Parameters (millions)	Encoding					
		No Encoding		***Quantum***		***Random Projection***	
		Mortality	*Gender*	*Mortality*	*Gender*	*Mortality*	*Gender*
1	1.25	0.769 ± 0.004	**0.858** ± 0.002	**0.739** ± 0.003	**0.662** ± 0.003	0.657 ± 0.004	**0.591** ± 0.004
2	2.51	**0.771** ± 0.003	0.856 ± 0.004	0.738 ± 0.005	0.662 ± 0.005	**0.659** ± 0.003	0.588 ± 0.005
4	5.01	0.766 ± 0.004	0.853 ± 0.003	0.738 ± 0.004	0.659 ± 0.003	0.658 ± 0.005	0.589 ± 0.004
8	10.1	0.767 ± 0.003	0.855 ± 0.003	0.736 ± 0.003	0.661 ± 0.004	0.655 ± 0.004	0.588 ± 0.003
16	20.21	0.765 ± 0.003	0.852 ± 0.003	0.735 ± 0.004	0.657 ± 0.003	0.657 ± 0.004	0.587 ± 0.004

This shows that larger models are not always better in healthcare informatics (non-textual) as they often overfit, and these observations are consistent with widespread use of “comparatively” smaller deep models in this domain.

Supplementary Note 15 - Impact of complex MLPs on latent prediction tasks

To analyse impact of complexity of the latent prediction model, we perform latent gender prediction tasks on best performing Transformers and ViT trained on MIMIC-III, PhysioNet and eICU datasets (discussed above). We varied the number of hidden layers from 1 to 5, each having 128 nodes and followed by ReLU activations. Apart from the depth of MLP, we didn’t change any experimental setting described in the manuscript. Again, early stopping and model checkpoints were used to select the best performing model configuration on validation examples.

Supplementary Table S7: Impact on MLP complexity on the latent gender prediction from MIMIC-III transformer.

Depth	MIMIC-III		
	No Encoding	Quantum	Random Projection
1	0.709 ± 0.003	**0.551** ± 0.005	**0.523** ± 0.004
2	**0.711** ± 0.002	**0.551** ± 0.003	0.521 ± 0.002
4	0.704 ± 0.004	0.55 ± 0.002	**0.523** ± 0.003
5	0.706 ± 0.003	0.55 ± 0.003	0.522 ± 0.005

Supplementary Table S8: Impact on MLP complexity on the latent gender prediction from PhysioNet transformer.

Depth	PhysioNet		
	No Encoding	Quantum	Random Projection
1	**0.756** ± 0.002	0.578 ± 0.002	**0.589** ± 0.004
2	0.753 ± 0.004	0.577 ± 0.004	**0.589** ± 0.002
4	0.754 ± 0.003	**0.579** ± 0.003	0.585 ± 0.006
5	0.751 ± 0.003	0.574 ± 0.005	0.587 ± 0.003

Supplementary Table S9: Impact on MLP complexity on the latent gender prediction from eICU transformer.

Depth	eICU		
	No Encoding	Quantum	Random Projection
1	**0.908** ± 0.003	0.701 ± 0.003	0.589 ± 0.004
2	0.906 ± 0.004	0.689 ± 0.003	0.588 ± 0.003
4	0.905 ± 0.002	**0.702** ± 0.002	0.589 ± 0.006
5	0.906 ± 0.003	0.688 ± 0.003	**0.591** ± 0.002

Tables S7, S8 and S9 illustrate that no significant improvement in performance of latent prediction from Transformers is observed on increasing the number of hidden layers in these MLPs. Again, similar behaviour is observed for ViTs (Tables S10, S11 and S12). The general trends of encoded data being worse than the standard ones in retaining input characteristics are still maintained. Again, we would like to point out that increasing the depth or complexity of MLPs resulted in overfitting and didn't translate to improved predictive performance.

Supplementary Table S10: Impact on MLP complexity on the latent gender prediction from MIMIC-III ViT.

Depth	MIMIC-III		
	No Encoding	Quantum	Random Projection
1	**0.672** ± 0.004	0.549 ± 0.003	0.489 ± 0.004
2	0.671 ± 0.003	**0.551** ± 0.004	**0.491** ± 0.002
4	0.67 ± 0.004	0.547 ± 0.004	**0.491** ± 0.004
5	0.671 ± 0.004	0.548 ± 0.003	0.488 ± 0.003

Supplementary Table S11: Impact on MLP complexity on the latent gender prediction from PhysioNet ViT.

Depth	PhysioNet		
	No Encoding	Quantum	Random Projection
1	**0.719** ± 0.004	**0.559** ± 0.003	0.554 ± 0.005
2	0.716 ± 0.003	**0.559** ± 0.003	0.552 ± 0.003
4	0.718 ± 0.004	0.556 ± 0.003	**0.556** ± 0.006
5	0.717 ± 0.005	0.557 ± 0.004	0.554 ± 0.003

Supplementary Table S12: Impact on MLP complexity on the latent gender prediction from eICU ViT.

Depth	eICU		
	No Encoding	Quantum	Random Projection
1	**0.873** ± 0.004	**0.663** ± 0.004	**0.591** ± 0.002
2	**0.873** ± 0.003	0.661 ± 0.003	0.59 ± 0.003
4	0.871 ± 0.004	0.662 ± 0.002	0.588 ± 0.006
5	0.87 ± 0.005	0.662 ± 0.004	0.589 ± 0.003

References

[1] Harutyunyan, H., Khachatrian, H., Kale, D.C., Ver Steeg, G., Galstyan, A.: Multitask learning and benchmarking with clinical time series data. Scientific data **6**(1), 1–18 (2019)

[2] Tang, S., Davarmanesh, P., Song, Y., Koutra, D., Sjoding, M.W., Wiens, J.: Democratizing ehr analyses with fiddle: a flexible data-driven preprocessing pipeline for structured clinical data. Journal of the American Medical Informatics Association **27**(12), 1921–1934 (2020)
